# Exosomal miRNAs as Potential Biomarkers to Monitor Phosphodiesterase 5 Inhibitor Induced Anti-Fibrotic Effects on CCl_4_ Treated Rats

**DOI:** 10.3390/ijms22010382

**Published:** 2020-12-31

**Authors:** Andre Broermann, Ramona Schmid, Ogsen Gabrielyan, Marlene Sakowski, Claudia Eisele, Sascha Keller, Michael Wolff, Patrick Baum, Birgit Stierstorfer, Jochen Huber, Bernhard K. Krämer, Berthold Hocher, Ruediger Streicher, Denis Delić

**Affiliations:** 1 Cardiometabolic Diseases Research, Boehringer Ingelheim Pharma GmbH & Co. KG, Birkendorferstr.65, 88397 Biberach, Germany; andre.broermann@boehringer-ingelheim.com (A.B.); ruediger.streicher@boehringer-ingelheim.com (R.S.); 2 Translational Medicine & Clinical Pharmacology, Boehringer Ingelheim Pharma GmbH & Co. KG, Birkendorferstr.65, 88397 Biberach, Germany; ramona.schmid@boehringer-ingelheim.com (R.S.); ogsen.gabrielyan@boehringer-ingelheim.com (O.G.); marlene.sakowski@boehringer-ingelheim.com (M.S.); claudia.eisele@boehringer-ingelheim.com (C.E.); michael.wolff@boehringer-ingelheim.com (M.W.); patrick.baum@boehringer-ingelheim.com (P.B.); 3 Drug Metabolism & Pharmacokinetics, Boehringer Ingelheim Pharma GmbH & Co. KG, Birkendorferstr.65, 88397 Biberach, Germany; sascha.keller@boehringer-ingelheim.com; 4 Drug Discovery Sciences, Boehringer Ingelheim Pharma GmbH & Co. KG, Birkendorferstr.65, 88397 Biberach, Germany; birgit.stierstorfer@boehringer-ingelheim.com; 5 Clinical Operations, Boehringer Ingelheim Pharma GmbH & Co. KG, Birkendorferstr.65, 88397 Biberach, Germany; jochen.huber@boehringer-ingelheim.com; 6 Fifth Department of Medicine (Nephrology/Endocrinology/Rheumatology), University Medical Centre Mannheim, University of Heidelberg, 68167 Mannheim, Germany; Bernhard.Kraemer@umm.de (B.K.K.); Berthold.Hocher@medma.uni-heidelberg.de (B.H.); 7 Key Laboratory of Study and Discovery of Small Targeted Molecules of Hunan Province, School of Medicine, Hunan Normal University, Changsha 410078, China

**Keywords:** liver fibrosis, CCl_4_, PDE 5 inhibitor, gene expression, microRNAs, exosomes

## Abstract

MicroRNAs (miRNAs) are short, non-coding RNA species that are important post-transcriptional regulators of gene expression and play an important role in the pathogenesis of non-alcoholic fatty liver disease. Here, we investigated the phosphodiesterase 5 (PDE5) inhibitor induced effects on hepatic and plasma exosomal miRNA expression in CCl_4_-treated rats. In the present study, hepatic miRNA profiling was conducted using the Nanostring nCounter technology and mRNA profiling using RNA sequencing from PDE5 treated rats in the model of CCl_4_-induced liver fibrosis. To evaluate if the PDE5 inhibitor affected differentially expressed miRNAs in the liver can be detected in plasma exosomes, qRT-PCR specific assays were used. In livers from CCl_4_-treated rats, the expression of 22 miRNAs was significantly increased (>1.5-fold, adj. *p* < 0.05), whereas the expression of 16 miRNAs was significantly decreased (>1.5-fold, adj. *p* < 0.05). The majority of the deregulated miRNA species are implicated in fibrotic and inflammatory processes. The PDE5 inhibitor suppressed the induction of pro-fibrotic miRNAs, such as miR-99b miR-100 and miR-199a-5p, and restored levels of anti-fibrotic miR-122 and miR-192 in the liver. In plasma exosomes, we observed elevated levels of miR-99b, miR-100 and miR-142-3p after treatment with the PDE5-inhibitor compared to CCl_4_/Vehicle-treated. Our study demonstrated for the first time that during the development of hepatic fibrosis in the preclinical model of CCl_4_-induced liver fibrosis, defined aspects of miRNA regulated liver pathogenesis are influenced by PDE5 treatment. In conclusion, miRNA profiling of plasma exosomes might be used as a biomarker for NASH progression and monitoring of treatment effects.

## 1. Introduction

Non-alcoholic fatty liver disease (NAFLD) describes a spectrum of disease that ranges from simple steatosis to nonalcoholic steatohepatitis (NASH), which may lead to liver cirrhosis and hepatocellular cancer (HCC). NAFLD has become the most common chronic liver disorder in Europe [1] and the US [2]: the prevalence is estimated to be 20% to 30% in the general population. NAFLD is recognized as the hepatic manifestation of the metabolic syndrome and type 2 diabetes mellitus, while NASH with sustained inflammation and chronic liver injury represents the progressive form towards liver fibrosis, cirrhosis and liver failure [3]. To date, the most common non-invasive laboratory abnormality found in patients with NAFLD is a mild to moderate elevation of serum aminotransferases ALT and AST [4]. Recent studies suggest that ALT and AST are less precise predictors of NAFLD [5,6]. Liver biopsy is the current gold standard in diagnosis and prognosis; nevertheless, it is an expensive and invasive procedure with high risk of sampling error and risk of complications including pain, bleeding, and, in very rare cases, death [7]. For these reasons, there is an unmet need to identify biomarkers which reflect early effects during disease development and progression and which potentially allow monitoring predictive and/or prognostic treatment effects.

Hepatic stellate cells (HSCs) have been recognized as the major source of pathologically increased extracellular matrix under diseased conditions [8]. HSC activation and trans-differentiation into myofibroblasts is believed to be one of the key events in the process of liver fibrosis [9]. Epithelial to mesenchymal transition and matrix production by HSCs is predominantly mediated by transforming growth factor beta (TGF-β) [10]. Several miRNA were reported in the regulation of TGF-β mediated HSC activation.

MicroRNAs (miRNAs) are small, 18–22 nucleotides in length, non-coding RNA molecules that modulate differentiation, growth, apoptosis and proliferation of cells by interfering with protein synthesis by either inducing mRNA degradation or repressing translation [11]. miR-122 expression is enriched in the liver and represents more than 50% of the hepatic miRNome [12,13] and has metabolic, anti-inflammatory, and anti-tumorigenic functions in the liver [14]. Upon liver injury/damage, miR-122 is released into circulation [15] and the estimated half-life is approximately 24 h [16,17]. miR-122 expression is altered in various liver diseases, such as hepatitis C virus infection (HCV) [18], drug induced liver injury (DILI) [19], HCC [20,21], primary biliary cirrhosis [22], alcoholic steatohepatitis [23] and NASH [24]. miR-122 is considered to be a highly sensitive and specific biomarker in blood reflecting hepatocyte injury as increased levels of miR-122 were described for HCV infection [25,26], HCC [27], DILI [28], alcoholic liver disease [29], and NASH [30]. In NASH patients and in animal models circulating, miR-122 levels are elevated in serum whereas its expression is down-regulated in liver tissue [24,31].

Exosomes are cup-shaped 40–100 nm membrane vesicles that derive from the multivesicular bodies in the endocytic compartment. Exosomal cargo, including miRNAs can be delivered to distant target cells, which is increasingly being recognized as an important mode of cell-cell communication; in addition, a potential source of novel non-invasive biomarkers for liver diseases [32]. In contrast to free miRNAs, exosomal miRNAs are remarkably stable as they are protected from endogenous RNase activity. Recent studies have demonstrated that primary and immortalized hepatocytes are capable to release exosomes [33,34,35]. Furthermore, exosome release is increased during accumulation of lipotoxic lipids in hepatocytes, which is a key mechanism of liver injury and disease progression in NASH [34]. The liver is an important source of exosomes, and exosomal miR-122 content is enriched in blood in NAFLD animals [36]. Therefore, the exosomal content could provide valuable insights into hepatic pathophysiology.

To date, no approved therapy for liver fibrosis or effective disease modifying regimen for NASH is currently available, despite its high unmet medical need. Pharmacologic interventions to improve the metabolic syndrome including lipid lowering medications (statins, fibrates, polyunsaturated fatty acids, antioxidants (vitamin E, vitamin C, betaine, probucol) anti-TNF agents, ursodeoxychilc acid, insulin sensitizers (metformin, thiazolidinediones) or anti-diabetic drugs (e.g., DPP-4 inhibitors and SGLT2 inhibitors) are currently used. The advanced clinical development pipeline in NASH has faced quite significant attrition (e.g., inhibitors for ASK-1, LOX-L2 or caspases). Up to date the majority of clinical development programs in NASH did not result in the expected efficacy on primary endpoints or faced tolerability issues. The interim data of the currently running Phase III clinical trials for Obeticholic Acid (Intercept) and Elafibranor (Genfit) also indicate that it is still open whether these mechanisms have a risk-benefit profile that will be supportive for approval and whether they would significantly improve the NASH phenotype in patients. If the other mechanisms that are being investigated in Phase III clinical trials in NASH, Resmetirom (Madrigal) or Cenicriviroc (Allergen) will be able to demonstrate meaningful effects on NASH resolution and fibrosis reduction is also still open [37,38].

cGMP-PKG pathway plays a central role in fibrotic processes and defects in cGMP-PKG pathway contribute to impaired NO-dependent responses in hepatic stellate cells upon activation [39]. cGMP levels can be modulated in different ways: soluble guanylate cyclase stimulators or activators or phosphodiesterase inhibitors, such as PDE5 inhibitor. In a previous study it was demonstrated that praliciguat, a sGC stimulator, suppresses stellate cell activation and inhibits both fibrosis and inflammation in preclinical models [40,41]. In another study it has been shown that the sGC stimulator riociguat reduces fibrogenesis and portal pressure in cirrhotic rats [42]. Therefore, pharmacological interventions that affect the NO-sGC-cGMP pathway are promising drugs for decreasing both inflammation and fibrosis in patients with NASH.

In this study, we use the chronic CCl_4_ rat model, which resembles all important properties of human liver fibrosis, including inflammation, regeneration, fiber formation and fibrosis progression [43]. Novel findings were identified after the intervention with a PDE5 inhibitor which prevented the CCl_4_-induced changes in distinct miRNA species whose expression was reflected in plasma exosomal miRNA expression. Our results suggest that monitoring of plasma exosomal miRNA expression might reflect disease progression and is a potential treatment effect marker for PDE5 inhibitor induced anti-fibrotic effects.

## 2. Results

### 2.1. Experimental Design

The aim of this study was to identify the effects of the PDE5 inhibitor on miRNAs differentially expressed in livers from CCl_4_-treated rats and to assess whether these effects can be accurately reflected in plasma exosomes. As a first step, large-scale miRNA and mRNA expression profiles from the same liver tissue samples were evaluated using the Nanostring and RNA sequencing platforms, respectively. Changes in miRNA expression level were identified by moderated t-test evaluating only those miRNAs with at least 1.5-fold expression changes at an adjusted *p*-value < 0.05. Functional annotations of these miRNAs were searched in several databases including miRWalk (http://www.umm.uni-heidelberg.de/apps/zmf/mirwalk), TargetScan (http://www.targetscan.org), miRBase (http://www.mirbase.org), and PubMed (http://www.ncbi.nlm.nih.gov/pubmed). Inverse correlated miRNA-target mRNA pairs were identified using Ingenuity Pathway Analysis (http://www.qiagenbioinformatics.com). In the next step quantitative real-time PCR was used to detect differentially expressed hepatic miRNAs which were significantly affected after PDE5 inhibitor treatment in plasma exosomes.

### 2.2. Effects of PDE5 Inhibitor on Body Weight, Liver Weight, Plasma Alanine Aminotransferase and Hepatic cGMP Level in CCl_4_-Induced Liver Injury

Before administration of the PDE5 inhibitor and at study termination, body weights of CCl_4_-treated rats and control animals were comparable (Table 1). At study termination, CCl_4_-treated rats displayed signs of liver injury, characterized by elevated plasma ALT levels (330.7 U× L^−1^; *p* < 0.05) and increased liver weights (21.4 g; *p* < 0.05) compared to control animals (ALT = 58.2 U× L^−1^; liver weight = 16.8 g) (Table 1). Exposure of CCl_4_-treated rats to PDE5 inhibitor did not result in significant changes in body weight, liver weight or plasma ALT levels (Table 1). Target engagement was detected by cGMP measurements. PDE5 inhibitor treatment resulted in significantly increased hepatic cGMP level compared to Vehicle- and CCl_4_-treatment (Table 1). Moreover, no significant effects on metabolic parameters (plasma trigylycerides and plasma cholesterol) were observed after treatment with the PDE5 inhibitor in CCl_4_ treated rats (data not shown).

### 2.3. Effects of PDE5 Inhibitor on CCl_4_-Treated Rats on Markers of Fibrosis and Inflammation

**Figure 1:**
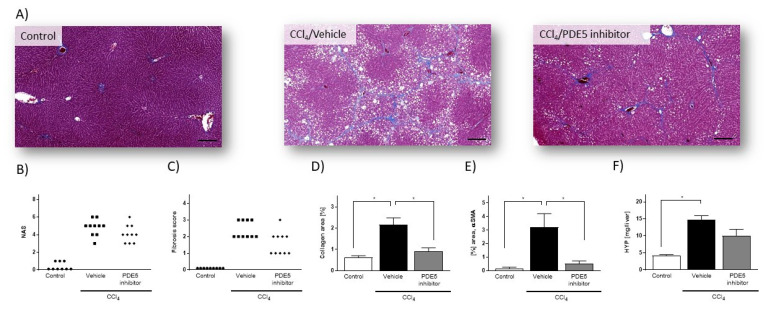
Histology assessment of liver sections from control rats, from rats with CCl_4_-induced liver injury and from CCl_4_/PDE5 inhibitor treated rats. (**A**) Representative images of Masson’s trichrome stain (Masson). **(B**) NAFLD activity score (NAS). (**C**) Fibrosis scores. Data in (**B**) and (**C**) represent scores of individual animals (*n* = 9–10). (**D**) Image-based quantification of collagen-positive area in Masson’s trichrome stained liver sections. (**E**) Hepatic αSMA content. (**F**) Hepatic HYP content. Data in (**D**–**F**) represent mean (*n* = 5) ± SEM, * *p* < 0.05; scale bar: 100µm.

Histopathological investigation of liver from rats treated with CCl_4_ presented signs of tissue injury and pronounced fibrosis (Figure 1A). In CCl_4_-treated rats, NAS ranged from 3 to 6 (4.8 ± 0.29) (Figure 1B) and fibrosis scores from 2 to 3 (2.4 ± 0.16) (Figure 1C). Quantitative histological assessment based on Masson’s trichrome staining revealed collagen-positive area of 2.16 ± 0.31% in CCl_4_-treated rats, as compared with 0.61 ± 0.08% in control rats (Figure 1D). Exposure of CCl_4_-treated rats to PDE5 inhibitor did not reveal significant changes in NAS (PDE5 inhibitor: 4.1 ± 0.31) (Figure 1B) and fibrosis score (PDE5 inhibitor: 1.6 ± 0.22) (Figure 1C). PDE5 inhibitor treatment resulted in a significant reduction of collagen-positive area (0.91 ± 0.15) in CCl_4_-treated rats compared to Vehicle-treated CCl_4_-treated rats (Figure 1D). In CCl_4_-treated rats αSMA protein levels were 22-fold significantly higher (*p* < 0.05; Figure 1E) than in control animals. PDE5 inhibitor treatment significantly attenuated αSMA protein levels in CCl_4_-treated rats (0.52 ± 0.19% area) compared to CCl_4_/Vehicle-treated rats (3.22 ± 0.98% area) (Figure 1E). The hepatic hydroxyproline content was 3.3-fold significantly higher (*p* < 0.05; Figure 1F) in CCl_4_-treated rats compared to Vehicle-treated CCl_4_-treated rats whereas administration with the PDE5 inhibitor did not result in a significant change.

### 2.4. Effects of PDE5 Inhibitor on Differentially Expressed miRNAs in Livers from CCl_4_ Treated Rats

**Figure 2:**
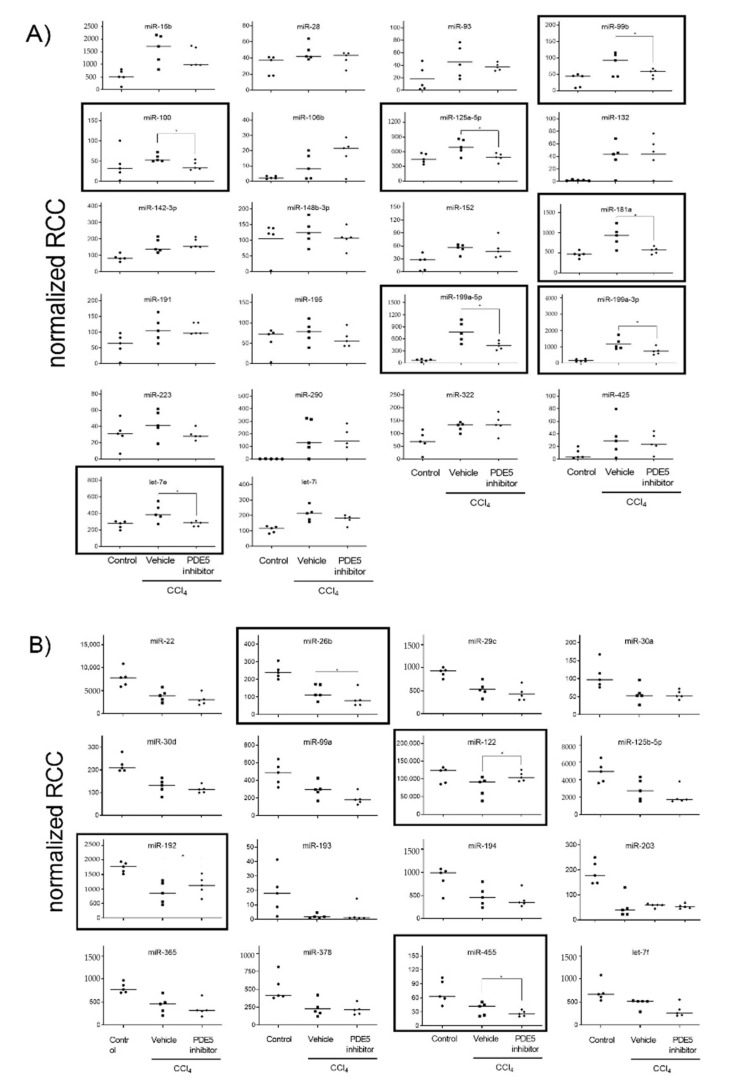
Up-regulated (**A**) and down-regulated (**B**) hepatic miRNA expression. MiRNA levels of control, CCl_4_/Vehicle and CCl_4_/PDE5 inhibitor treated rats revealed by Nanostring analysis. Absolute values are displayed for each animal by normalized reporter cell counts (RCC). MiRNAs whose expression is significantly changed in one of the treatment groups are highlighted with boxes. * *p* < 0.05.

**Figure 3:**
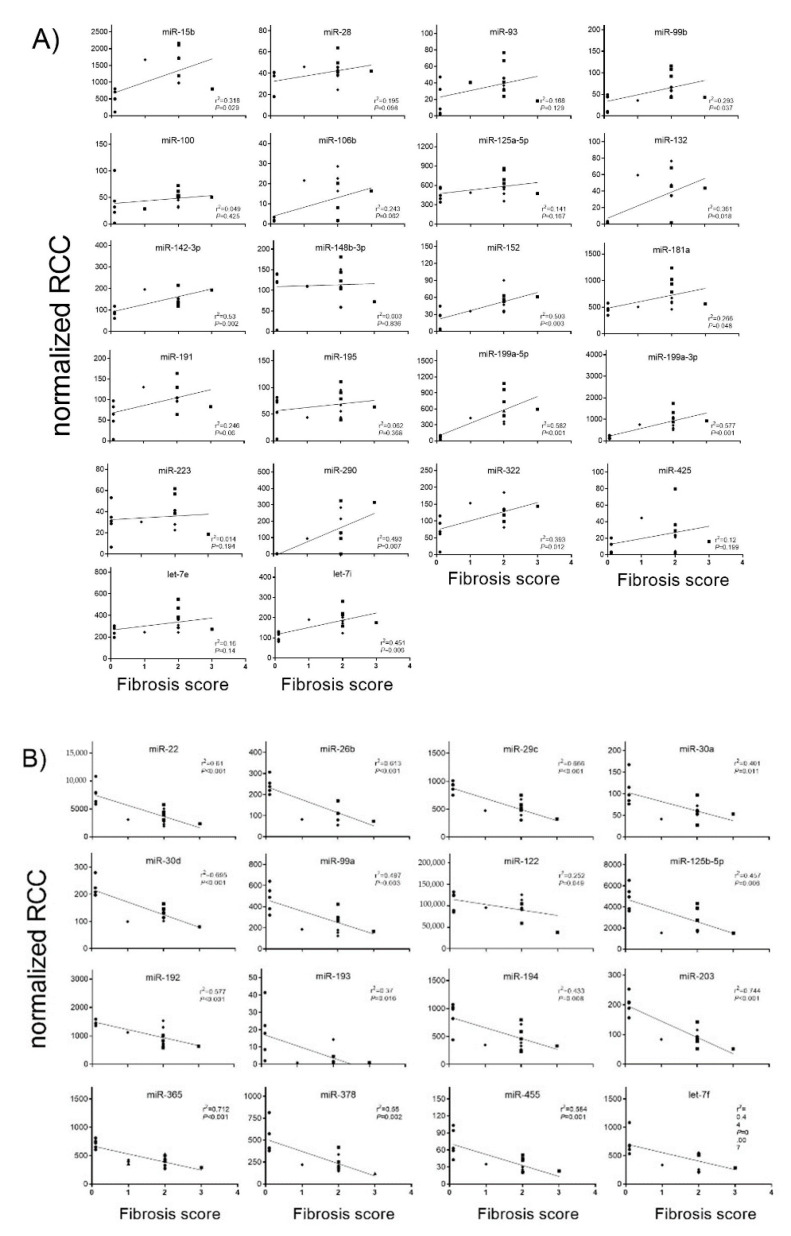
Correlation of fibrosis scores with up-regulated hepatic (**A**) and down-regulated (**B**) miRNA expression. MiRNA levels of control, CCl_4_/Vehicle and CCl_4_/PDE5 inhibitor treated rats were correlated with fibrosis scores. Absolute values are displayed for each animal by normalized reporter cell counts (RCC). Spearman’s correlation coefficient and corresponding *p*-values are indicated.

Principal component analysis revealed that the miRNA expression profiles were different between control and CCl_4_-treated groups (Figure S1). Compared with control rats, in CCl_4_/Vehicle-treated rats, the hepatic expression of the 22 miRNAs miR-15b, miR-28, miR-93, miR-99b, miR-100, miR-106b, miR-125a-5p, miR-132, miR-142-3p, miR-148b-3p, miR-152, miR-181a, miR-191, miR-195, miR-199a-5p, miR-199a-3p, miR-223, miR-290, miR-322, miR-425, let-7e and let-7i was significantly up-regulated (Figure 2A), whereas the hepatic expression of the 16 miRNAs miR-22, miR-26b, miR-29c, miR-30a, miR-30d, miR-99a, miR-122, miR-125b-5p, miR-192, miR-193, miR-194, miR-203, miR-365, miR-378, miR-455 and let-7f was significantly down-regulated (Figure 2B). PDE5 inhibitor significantly ameliorated the expression of miR-99b, miR-100, miR-125a-5p, miR-181a, miR-199a-5p, miR-199a-3p and let-7e compared to the CCl_4_/Vehicle-treated group, respectively (Figure 2A). Significant restorative effects on the expression of miR-122 and miR-192 in CCl_4_/PDE5 inhibitor-treated rats compared to CCl_4_/Vehicle-treated rats whereas the PDE5 inhibitor treatment led to a further decrease in the hepatic expression of miR-26b and miR-455 compared to CCl_4_/Vehicle-treated. Increased levels of miR-15b, miR-99b, miR-132 miR-142-3p, miR-152, miR-181a, miR-199a-5p, miR-199a-3p, miR-290, miR-322 and let-7i are characterized by a significant (*p* < 0.05) positive correlation with the fibrosis score (Figure 3A). Decreased levels of miR-22, miR-26b, miR-29c, miR-30a, miR-30d, miR-99a, miR-122, miR-125b-5p, miR-192, miR-193, miR-194, miR-203, miR-365, miR-378, miR-455 and let-7f showed a significant (*p* < 0.05) negative correlation with fibrosis score (Figure 3B). The functions of the deregulated miRNAs based on published data are summarized in Table 2. Most of the deregulated miRNAs in the liver from CCl_4_-treated rats were involved in fibrotic processes, such as the miR-29c, miR-30a, miR-181a, miR-191, miR-192, miR-193, miR-194, miR-199a-5p, miR-199a-3p, miR-378 and miR-425 (Table 2). The translatability into observations on deregulated miRNA expression in humans is listed in Table 2.

### 2.5. Integrated miRNA-mRNA Analysis

To identify target mRNAs of the 38 differentially expressed miRNAs in livers from CCl_4_/Vehicle-treated rats, RNA was sequenced from the liver tissues of all experimental groups. All up- and down-regulated (1.5-fold; adj. *p* <  0.05) genes are summarized in Supplementary Tables S1 and S2. In total, the expression of 2295 genes is deregulated (1.5-fold; adj. *p* < 0.05) in CCl_4_/Vehicle-treated rats compared to the control rats: the expression of 1782 genes is significantly up-regulated (Supplementary Table S1) whereas the expression of 513 genes is significantly down-regulated (Supplementary Table S2).

**Figure 4:**
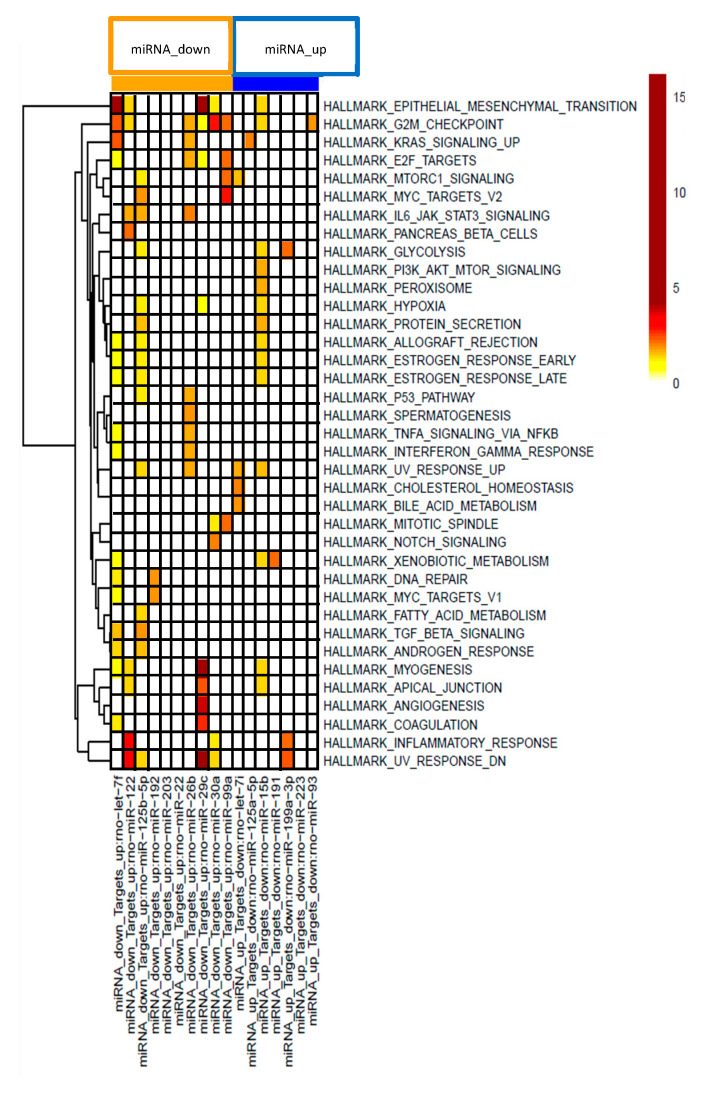
Gene set enrichment analysis of miRNA-mRNA pairs according to 37 hallmark pathways. Effects on distinct pathways are represented by colors indicating enrichment scores.

To further characterize the effects of the PDE5 inhibitor on mRNA-miRNA interactions in CCl_4_ -treated rats pathway analysis was performed using MSigDB [44]. Hypergeometric testing using the hallmark gene set from MSigDB revealed that the down-regulated miRNAs correspond to up-regulated target mRNAs that are involved in fibrotic pathways, such as epithelial mesenchymal transition and inflammatory processes, such as TNFα signaling, IL-6/JAK/STAT3 signaling or IFN signaling (Figure 4). Down-regulated mRNAs that correspond to up-regulated miRNAs are involved in oxidative stress and metabolic pathways (Figure 4).

### 2.6. Effects of PDE5 Inhibitor on Plasma Exosomal miRNA Level

**Figure 5:**
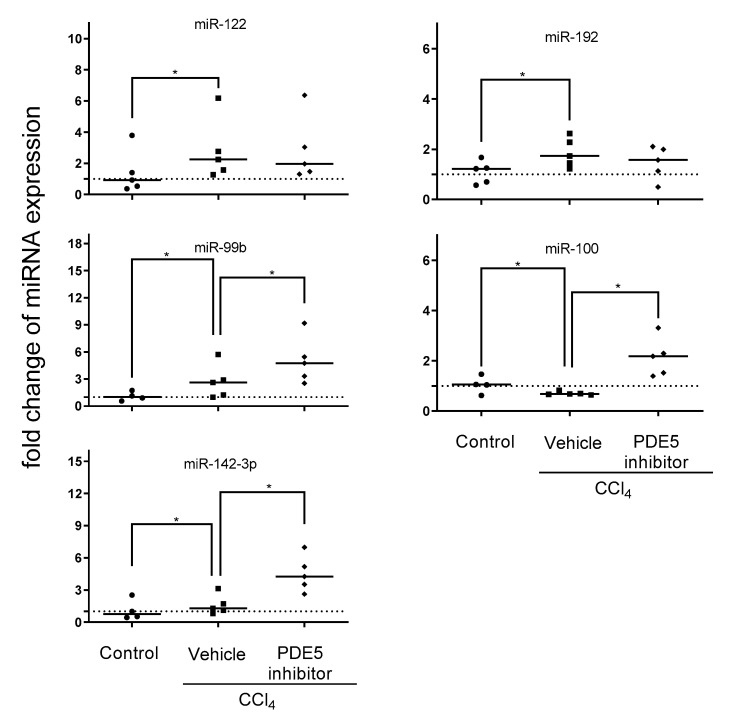
Plasma exosomal miRNA expression. Plasma exosomal levels of miR-99b, miR-100, miR-142-3p, miR-122, and miR-192 in control, CCl_4_/Vehicle and CCl_4_/PDE5 inhibitor treated rats. Relative plasma exosomal miRNA expression analyzed by qRT-PCR was normalized to the mean expression of control rats. The median is depicted and significant differences between experimental groups are indicated by * (*p* < 0.05).

To assess which of the aforementioned treatment affected miRNA expression profiles can be reflected in plasma exosomes qRT-PCR was employed to address effects of PDE5 inhibitor affected miRNA expression in liver of miR-99b, miR-100, miR-122, miR-142-3p, miR-192, miR-199a-5p and miR-455. CCl_4_ treatment led to significantly increased levels of miR-122, miR-99b and miR-192 and significantly decreased level of miR-100. Plasma exosomal miRNA analysis revealed significantly enhanced levels of miR-99b, miR-100 and miR-142-3p after PDE5 inhibitor treatment while Vehicle-treated rats showed no significant change in exosomal miR-142-3p expression (Figure 5). The expression analyses of miR-199a-5p and miR-455 were characterized by below level of quantification. Correlation analysis revealed a significant positive correlation between plasma exosomal miR-122 and ALT level (Figure S2).

## 3. Discussion

The present study shows for the first time that the PDE5 inhibitor induced effects on hepatic miRNA and their corresponding target mRNA expression in CCl_4_-treated rats. Integrating both hepatic miRNA and mRNA expression profiles enriched pathways, which are deregulated in CCl_4_-treated rats, such as fibrotic pathways including epithelial mesenchymal transition and inflammatory processes, such as TNFα signaling and IL-6/JAK/ STAT3 signaling. In particular, the PDE5 inhibitor exerts its strongest effects on fibrotic processes.

miR-122 is predominantly expressed in the liver and plays multifunctional roles in the regulation of lipid metabolism, cell cycle, and in hepatitis C virus replication [45,46,47]. In various NAFLD animal models, decreased level of hepatic miR-122 were reported whose degree of deregulation correlated with disease severity which also coincides with increased miRNA-122 level in the circulation [30,48]. This inverse correlation was also observed in human studies [26,31,49,50]. Indeed, higher levels of miR-122 were detected in serum of patients with NAFLD compared to healthy controls and positively correlated with disease severity [26,49] which was also observed in extracellular vesicles [36]. In addition, increased serum miR-122 level correlates with increased ALT level in NASH patients [51]. miR-122 expression was found to be concentrated at the boundary of hepatocyte wall rather than being distributed throughout the cytoplasm and being immediately released upon liver injury leading to elevated levels in the circulation. Our study revealed correlation of increased plasma miR-122 level with increased ALT level. Ameliorated hepatic miR-122 expression was observed after PDE5 inhibitor treatment in CCl_4_ treated rats. Integrated miR-122/target mRNA analysis revealed a significant enrichment in inflammatory and fibrotic pathways in CCl_4_ treated rats, which were attenuated after PDE5 inhibitor treatment. A key target gene of miR-122 is NUMB which is known to negatively influence the NOTCH pathway, and the associated regulation of cell fate has drawn attention to the potential role of NUMB in tumorigenesis in a number of solid tumors [52]. Up-regulated NUMB expression which might have resulted from decreased miR-122 level is slightly affected after treatment with PDE5 inhibitor.

The expression of miR-192, a pro-fibrotic miRNA involved in the TGF-β signaling pathway, is mainly apparent in quiescent hepatic stellate cells (HSC) and found to be down-regulated in liver fibrosis mouse models and in human cirrhotic livers [53]. Significant increases in miR-192 level were detected in the serum of subjects with NASH compared to healthy controls [54]. Our study revealed a significant negative correlation with the fibrosis score. Functional experiments confirmed a protective effect of down-regulation of miR-192-5p on hepatocytes, suggesting a role of miR-192-5p in limiting liver injury. One potential target gene is Zeb2 (Zinc finger E-box-binding homeobox 2), an important regulator of EMT, as a potential target gene mediating the function of miR-192-5p [55]. Our data suggest that in contrast to miR-192 the expression of ZEB2 is not affected after treatment. ZEB2 is also regulated by TGF-β and other miRNA species such as members of the miR-200 family which are not affected in our fibrosis model [56].

In a previous study, it was shown that miR-142-3p is implicated in the TGF-β signaling pathway and that its expression is down-regulated upon activation of HSCs [57]. Intriguingly, we observed that in livers of CCl_4_ treated rats the expression of miR-142-3p is up-regulated which implied that effects of TGF-β on miR-142-3p might vary among different hepatic cells or might be a consequence of negative feedback loop networks. The hepatic expression level showed a significant positive correlation with fibrosis score, and in plasma, we observed that increased exosomal miR142-3p level are associated with higher ALT level. In contrast, plasma miR-142-3p was markedly down-regulated in patients with hepatic cirrhosis [57]. Downregulation of miR-455-3p occurred in activated HSCs induced by TGF-β and is downregulated in different hepatic fibrosis models including CCl_4_ induced liver fibrosis which is in line with our results and a significant negative correlation was observed. Mechanistically, miR-455-3p regulated HSF expression by binding to the 3’UTR of its mRNA directly [58]. Our results demonstrated that the PDE5 inhibitor further alleviated hepatic miR-455-3p level which resulted in no further increase of HSF mRNA expression (Table S1).

Furthermore, the hepatic expression of the up-regulated pro-fibrotic miRNAs miR-199a-3p and miR-199a-5p is significantly attenuated after PDE5 inhibitor treatment in CCl_4_ administered rats. miR-199a-5p is up-regulated during the fibrogenic response to tissue injury and is a general marker for fibrogenesis in various animal models such as bleomycin-induced lung fibrosis, CCl_4_-induced liver fibrosis and unilateral ureteral obstruction model of kidney fibrosis [59,60]. Both miRNAs showed significant a positive correlation with fibrosis score.

Anti-fibrotic miRNAs which are known to be in involved in the regulation of EMT and are regulated in CCl_4_ rat model such as members of the miR-200 family were not deregulated after treatment in our experiment. In a previous report, it was shown that CCl_4_ mediated inhibition of miR-200a was enhanced after treatment with carvedilol [61]. Another important anti-fibrotic miRNA is represented by members of the miR-29 family. Moreover, it has been demonstrated that adenovirus-mediated expression of miR-29a can attenuate CCl_4_ induced liver fibrosis in mice [62]. We also observed a reduction of hepatic miR-29 expression after CCl_4_ administration but treatment with PDE5 inhibitor did not result in significantly restored levels. Nevertheless, it is worthwhile to monitor treatment effects of these well characterized anti-fibrotic miRNAs in human studies.

Expression changes of miRNAs involved in cell division, proliferation and differentiation of hepatocytes were observed in our study. In particular, increased expression of miR-99b in the liver [63] and increased expression in serum miR-100 [64] was found in patients with hepatocellular carcinoma. Our study revealed increased hepatic levels of miR-99b whereas both miRNAs miR-99b and miR-100 were significantly increased after PDE5 inhibitor treatment in plasma exosomes compared to Vehicle-treated rats. It has been observed that ZEB1 (Zinc Finger E-Box Binding Homeobox 1) is regulating the expression of the microRNA-99b/let-7e/microRNA-125a cluster. Indeed, our results revealed also a significant up-regulation of let-7e and miR-125a whose expression was not influenced upon PDE5 inhibitor treatment.

Recently, it was shown that the PDE5 inhibitor sildenafil induced hepatoprotection against CCl_4_ induced toxicity [65]. Anti-fibrotic effects of drugs influencing the NO/sGC/sGMP pathway such as the sGC stimulator riociguat were demonstrated in both acute and advanced bile duct ligation model and the acute CCl_4_ fibrosis model whereas no significant decreases in markers like hydroxyproline were also described [42]. In a previous study by Hall et al., 2018 a reduction in hydroxyproline was also not observed after treatment of CCl_4_ rats with the sGC stimulator praliciguat whereas α SMA was significantly reduced like in our study after PDE5 inhibitor treatment. In addition, we have observed a significant decrease in collagen area which further substantiates the anti-fibrotic effects after PDE5 inhibitor treatment. A limitation of our study is that we did not further dissect anti-fibrotic and anti-inflammatory effects of the PDE5 inhibitor in a more moderate fibrosis model like a diet-induced fibrosis rat model which is part of future investigation. In addition, it is also worthwhile to compare PDE5 inhibitor induced effects with sGC activator and/or sGC stimulator effects on hepatic and plasma exosomal miRNA expression in future studies. Phosphodiesterases in the liver are considered as therapeutic targets of cirrhotic hypertension [66] which should also be taken into account in future studies. Furthermore, we used the whole tissue for the screening of differentially expressed miRNAs and mRNAs. Future studies are required to further evaluate the molecular changes in a cell-type specific approach investigating hepatocytes, Kupffer cells and stellate cells separately. Furthermore, in depth molecular characterization including epigenetic analyses such as methylomics would increase the mechanistic understanding of the mode of action of drugs. Circulating microRNAs, in particular when packaged in extracellular vesicles, have shown great promise as biomarkers for various diseases. Moreover, a cell type specific approach, such as hepatocyte specific exosome enrichment, would identify the origin of liver injury and the effects of drugs which should also be considered in future studies. Finally, functional experiments using knock out animal experiments or therapeutic intervention of miRNA expression are considered in future to further dissect the linkage of PDE5 inhibitor treatment and subsequent effects on miRNA expression.

In human NAFLD/NASH studies, increased free-circulating levels of miR-15b, miR-28, miR-122, miR-132, miR-181a, miR-191, miR-192, miR-199a-5p and miR-199a-3p have been observed whereas decreased levels of miR-29c and miR-99a have been reported [67,68,69,70,71,72,73,74,75], which are in line with our findings in the liver of the CCl_4_-treated rats. Our observed inverse correlation of decreased hepatic miR-122 level and increased serum miR-122 level is consistent with previous reports in animal and human studies [14,31,47,76]. In a recent study, the localization of hybridization marks showed a consistent pattern of miR-122 expression at the edge of the wall of hepatocytes, suggesting that miR-122 is ready to be exported into the circulation [31]. Overall, these findings suggest that the lower expression of miR-122 in liver is a consequence of a high rate of release into the circulation rather than a down-regulation of miRNA expression. The observed inverse relationship between circulating and tissue expression of miR-122 seems to be the consequence of a dynamic regulation of the biology of miR-122 in the liver. Consequently, these dynamics of miR-122 production/release reflect the behavior of flux of substances/molecules between compartments [77]. In particular, PDE 5 inhibitor induced effects on the expression of miR-99b, miR-122 and miR-192 might be used in human studies for monitoring of treatment effects.

Several pharmacologic and non-pharmacologic interventions have been assessed to treat NASH, mainly targeting the metabolic disease, such as statins or anti-diabetic compounds. No approved therapy for liver fibrosis or effective disease modifying regimen for NASH is currently available, despite its high unmet medical need. miRNAs are also considered as therapeutic targets in different liver diseases. Miravirsen, an inhibitor of miR-122, is considered for treatment of hepatitis C virus [78]. The development of oligonucleotide-based miR-132 antagonists for the treatment of NASH is in its planning phase [79]. In general, interfering with miRNA expression could be an attractive target in order to develop new drugs that tackle the huge unmet medical need in NASH and liver fibrosis. A better understanding of the nature of plasma exosomes in NASH patients could also support the goal of replacing the need for biopsies, which is currently a huge burden for the patients and strongly affects the feasibility of clinical trials in NASH.

Our current study showed for the first time that anti-fibrotic effects induced by the PDE5 inhibitor are reflected by differentially expressed miRNAs in the liver and that some of these changes can be monitored in plasma exosomes, suggesting that miRNA profiling of plasma exosomes might be used as a useful biomarker for addressing effects during NASH progression and treatment with drugs which needs to be assessed in ongoing and future human studies.

## 4. Material and Methods

### 4.1. Animals

All animal care and experimental protocols were approved by the ethics review committee for animal experimentation of Boehringer Ingelheim Pharma GmbH & Co. KG. Animal studies are reported in compliance with the ARRIVE guidelines [80,81]. The carbon tetrachloride (CCl_4_) study was conducted at Boehringer Ingelheim (Biberach, Germany) and approved by local government authorities (Regierungspräsidium Tübingen, Germany) according to license 13-011-G (January 2015) and national animal welfare guidelines. Details on animals, experimental procedures, housing and husbandry as well as animals’ numbers are disclosed in the following methods sections.

### 4.2. Carbon Tetrachloride-Induced Hepatic Fibrosis in Rats

Male Sprague Dawley rats, 6–7 weeks of age (JANVIER LABS, Le Genest-Saint-Isle, France), were housed in pairs in a controlled environment (12 h light/dark cycle). All animals had ad libitum access to normal chow (KLIBA 3438; Provimi Kliba AG, Switzerland) and tap water. Animals received carbon tetrachloride (CCl_4_; 0.25 mL·kg^−1^) diluted in olive oil by p.o. administration three times a week for 8 weeks, and p.o. administration of Vehicle (0.5% natrosol) or PDE5 inhibitor (Tadalafil, Lilly Pharma, IN, USA) (7.5 mg·kg^−1^) twice daily while maintaining the CCl_4_ regimen. The schematic overview of the experimental design is depicted in Figure S3. Plasma was obtained by sublingual bleeding from isofluorane (2–3% in oxygen) anaesthetized animals. Depth of anesthesia was assessed by controlling reflexes (stimulated movement reflex, palpebral reflex, toe withdrawal reflex). At the end of the study, the animals were killed by final bleeding under pentobarbital anesthesia. Livers were weighed, and blood and liver samples were used for further analysis. 

### 4.3. Measurement of Plasma Alanine Aminotransferase

Plasma aminotransferase (ALT) activity was measured using 80 µL samples collected into EDTA tubes using a Cobas Integra 400 (Roche Diagnostics, Mannheim, Germany).

### 4.4. Measurement of cGMP Level in Liver Homogenate

In brief, 6 µL of 100 mM IBMX (phosphodiesteraseinhibitor) in DMSO was added together with 400 µL/100 mg liver 50 nM ^15^N_5_ cGMP in 2% H_3_PO_4_. The samples were homogenized in a TissueLyser for 10 min at 50 Hz/sec and then centrifuged for 30 min at ~ 20,000× *g*. The supernatant was centrifuged again for 15 min at ~20,000× *g*. To remove residual solid parts, the supernatant was transferred in an Amicon Ultra 30 K filter unit and centrifuged for 60 min at 14,000× *g*. The clear filtrate was completely used in the following process. cGMP was analyzed by liquid chromatography coupled to tandem mass spectrometry (LC-MS/MS) using ^15^N_5_ cGMP as an internal standard. The samples were subjected to solid phase extraction in the 96-well plate format followed by reversed-phase LC with gradient elution. The substance was detected and quantified by MS/MS using electrospray ionization in the positive ion mode.

All spectra and ion chromatograms are recorded on an AB Sciex Qtrap 5500 tandem mass spectrometer (AB Sciex, Darmstadt, Germany) equipped with a Turbo Spray ion source in positive electrospray mode. cGMP levels are reported as ratio of cGMP/^15^N_5_ GMP detector response.

### 4.5. Histology Assessment of Hepatic Fibrosis and αSMA Levels 

For histological analysis of the CCl_4_-treated animals, the right lobe of the liver was sectioned and fixed in phosphate-buffered 10% formaldehyde. Each formaldehyde-fixed sample was embedded in paraffin, cut into 4 µm-thick sections and stained with haematoxylin and eosin (H&E), Masson’s trichrome and with an antibody detecting αSMA (IHC) according to standard procedures. All slides were scored by the same pathologist using the NAFLD activity score (NAS) as described previously [82]. A semi-quantitative analysis of steatosis, lobular inflammation and hepatocellular ballooning was assessed using the H&E-stained sections. Fibrosis staging was performed on the Masson’s trichrome stained liver samples. For quantitative analysis of collagen positive area and αSMA positive area, histological slides were systematically scanned with a Zeiss AxioScan.Z1 slide scanner (Zeiss, Jena, Germany). Images were analyzed using a script based on HDevelop ImageAnalysis Toolbox (MVTec, München, Germany). Image segmentation was performed using texture and color information from color space transformation RGB to HSI and from color deconvolution. In the images, liver sections were segmented, and the area covered by liver segmented into mosaic tiles of size 1024 × 1024 pixels (from 500 to 1200 tiles per slide). For each tissue tile, the total area of tissue and the area with collagen rich tissue were detected and used to calculate a value describing fibrosis. For each tissue tile, the total area of tissue and the area with collagen rich tissue were detected and used to calculate a value describing fibrosis. In analogy, the area of macrosteatotic droplets, as well as the area positive for αSMA staining, was measured in order to evaluate the percentage of tissue affected with steatosis and the extent of αSMA expression per whole slide, respectively. The median value of all tiles was reported.

### 4.6. Biochemical Quantification of Hepatic Hydroxyproline Content 

Hepatic collagen content in samples from the CCl_4_ study was determined using 50–100 mg liver samples from two different lobes after hydrolysis in hydrochloric acid (6 M) for 16 h at 120 °C. Samples were cooled to room temperature and centrifuged at 18,000× *g* for 10 min. Standards and samples were transferred to 96-well plates and 50 µL of citrate-acetate buffer was added. After addition of chloramine T solution (100 µL), plates were incubated for 20 min at room temperature before the addition of Ehrlich’s reagent (100 μL; p-dimethylaminobenzaldehyde in ethanol:hydrochloric acid). Assay plates were incubated at 65 °C for 15 min and then cooled to room temperature. Sample absorbance was measured at 558 nm using a SpectraMax microplate reader (Molecular Devices, San Jose, CA, USA).

### 4.7. Total RNA Isolation

Five out of nine (Vehicle) or ten (CCl_4_ +/− PDE5 inhibitor-treatment) animals were selected according to their distribution across the median values in fibrotic scores for mRNA and miRNA expression analyses. Frozen liver tissue was homogenized with Precellys lysis with Precellys Steel 2.8 mm beads (PeqLab Biotechnology, Erlangen, Germany). For hepatic miRNA expression analyses, total RNA was isolated using the RNeasy Fibrous Tissue Mini Kit (QIAGEN, Hilden, Germany). Quality control and total RNA yield was quantified using the NanoDrop ND-1000 spectrophotometer (ThermoScientific, Wilmington, DE, USA).

### 4.8. Nanostring Analysis 

Total RNA (100 ng) was used to assess the miRNA expression using the nCounter Rat v1.5 miRNA CodeSet (based on miRBase v17, Nanostring Technologies, Seattle, WA, USA) which contains a library of 420 probes. The purified complexes were quantified on the nCounter Digital Analyzer and analyzed by nSolver software (v1.1; Nanostring Technologies, Seattle, WA, USA). The exact data analysis procedure can be found at: http://www.NanoString.com/media/pdf/MAN_nCounter_Gene_Expression_Data_Analysis_Guidelines.pdf.

### 4.9. mRNA Library Preparation, Sequencing and Data Analysis

RNA sequencing libraries were prepared using Illumina’s TruSeq RNA Sample Prep Kit-v2 (Illumina Inc., San Diego, CA, USA) according to the manufacturer’s instructions. The library concentrations were then quantified with the Quant-iT PicoGreen dsDNA Assay Kit (Quant-iT) using CLARIOstar (BMG LABTECH), and the library quality was determined by checking cDNA fragment size using a DNA1000 Kit on the Agilent Bioanalyzer 2100 (Agilent Tech Inc.) Libraries were then normalized to 2nM and subjected to cluster generation on a cBot system followed by single-read sequencing of 52 bp on an Illumina HiSeq4000 instrument (Illumina Inc.) The QC of the obtained reads was done with FASTQC v0.10.1. Based on RPKMs/FPKMs as obtained in the subsequent analysis (1.6.2), PCA and hierarchical clustering analysis were further used to identify outliers. Outliers were removed from subsequent analysis. Read processing was performed as previously described [83]. Fold changes and their respective significance were computed based on the read counts obtained for each gene using R and Bioconductor packages edgeR, DESeq2 or voom in conjunction with limma.

### 4.10. Integrated Analysis of miRNA and mRNA Expression

To further characterize the effects of PDE5 inhibitor on the effects of mRNA-miRNA interactions in CCl_4_-treated rats pathway analysis was performed using Hypergeometric testing using the hallmark gene set from MSigDB [44]. 

### 4.11. Exosomal miRNA Isolation

Exosomal miRNAs were isolated from 300 µL plasma using the exoRNeasy Serum/Plasma Maxi Kit (QIAGEN, Hilden, Germany) according to manufacturer’s instructions. Quality control was performed on the Agilent 2100 Bioanalyzer, using the RNA 6000 Nano Kit (Agilent Technologies Inc, Waldbronn, Germany) and miRNA concentration was measured using Quant-IT microRNA assay kit (Life Technologies, Carlsbad, CA, USA) according to manufacturer’s instructions.

### 4.12. Quantitative Real-Time PCR

miRNAs were reverse transcribed using TaqMan^®^ microRNA Reverse Transcription Kit (Life Technologies) and TaqMan miRNA assays (Life Technologies) specific for miR-99b, miR-100, miR-142-3p, miR-122, miR-192, miR-199a-5p, miR-455 and U6 snRNA as control. TaqMan^®^ gene expression master mix (Life Technologies) was used for the PCR reaction, which was performed according manufacturer’s protocol on a 7900HT real-time PCR System. All samples were run in duplicates and raw ct values were calculated using the SDS software v.2.4. Fold-change of expression was calculated using the comparative Ct method (2^−ΔΔct^) [84].

### 4.13. Statistical Analysis 

The data and statistical analysis comply with the recommendations on experimental design and analysis in pharmacology [85]. All data were analyzed using GraphPad Prism 7.00 software. Results are shown as individual values or as mean ± SEM or SD. Kruskal–Wallis was conducted on non-parametric NAS and fibrosis scores. One-way ANOVA with Tukey’s multiple comparison tests were used to evaluate statistical significance between control and treatment group data (* *p* < 0.05). In all experiments where Tukey’s multiple comparison tests were applied, F-test of the ANOVA demonstrated statistical significance (*p* < 0.05).

Differential expression was calculated applying the linear models approach incorporated in the limma software package using Tibco Spotfire version 6.5.2 (TIBCO Software, Palo Alto, Santa Clara, CA, USA). miRNAs > 1.5-fold and mRNAs > 1.5-fold differentially expressed between the compared groups with a *p*-value adjusted for multiple testing < 0.05 were considered to be significantly differentially expressed. Adjustment of *p*-values for multiple testing was done according to Benjamini and Hochberg. Graphical visualizations were generated via Tibco Spotfire (TIBCO Software).

## Figures and Tables

**Table 1:** Body weights, liver weights and plasma aminotransferase (ALT) and cGMP level in control, CCl_4_/Vehicle and CCl_4_/PDE_5_ inhibitor treated rats.

	No CCl_4_	CCl_4_ Vehicle	CCl_4_ PDE5 Inhibitor
Body weight at start of treatment [g]	305.1 ± 2.7	305.0 ± 2.6	304.9 ± 2.6
Body weight at study termination [g]	532.0 ± 11.3	505.3 ± 16.5	516.4 ± 16.0
Liver weight [g]	16.8 ± 0.6	21.4 ± 1.0 *	20.5 ± 1.1 *
ALT [U * L^−1^]	58.2 ± 2.4	330.7 ± 50.1 *	264.5 ± 58.3 *
cGMP [analyte counts/IS counts]	0.019 ± 0.002	0.019 ± 0.001	0.057 ± 0.004 *

Values represent mean ± SEM, *n* = 10 per experimental group; * *p* < 0.05; IS = internal standard.

**Table 2:** Effects of PDE5 inhibitor on differentially expressed miRNA in liver from CCl_4_ treated rats.

miRNA	CCl_4_ Vehicle/No CCl_4_	CCl_4_ PDE5 Inhibitor/CCl_4_ Vehicle	Functions in the Liver	PMID	Evidence in Human NAFLD/NASH Studies (Sample Matrix)	PMID
**Fibrosis**						
miR-199a-5p	11.7	−1.74 *	miR-199a-5p is upregulated during fibrogenic response to tissue injury. The progression of liver fibrosis is related with overexpression of the miR-199 family. Up-regulated miR-199a-5p inhibits nuclear receptor corepressor 1 translation in mice with NASH.	23459460 21283674 25269746	up (serum)	31754293
miR-199a-3p	7.36	−1.61 *	The progression of liver fibrosis is related with overexpression of the miR-199 family.	21283674	up (serum)	31754293
miR-425	3.38	1.07	miR-425-5p facilitates EMT and extracellular matrix degradation	28423650	no data	
miR-99b	3.05	−1.41 *	miR-99b contributes to promoting function in HCC migration and invasion through inhibiting KAI1 expression. miRNA-99b/let-7e/miRNA-125a cluster is highly-expressed in hepatocarcinoma, and its expression can be regulated by ZEB1.	29762829 30840268	no data	
miR-191	2.69	1.05	In patients with HCC, miR-191 was reported as a serum exosomal miRNA and a potential oncogenic target for HCC therapy. In HCC cancer cells, miR-191 was shown to be regulated mainly by DNA methylation and involved in the regulation of EMT.	31334580 21969817	up (serum)	32363318
miR-148b-3p	1.92	−1.17	Involved in hypoxia stress in human hepatic sinusoidal endothelial cells via NOX4 and eNOS/NO signaling; regulated by long non-coding RNA H19.	31082428	no data	
let-7i	1.9	−1.2	let-7i may control HCC tumorigenesis by regulating IGF1R directly and indirectly by interrupting the interplay between IGF1R and the IGF2BP.	27126374	no data	
miR-195	1.9	−1.24	miRNA-195 activates hepatic stem cells by targeting Smad7.	28929107	no data	
miR-142-3p	1.83	1.11	miR-142-3p repressed TGF-β-Smad signaling pathway to prevent HSCs activation through directly targeting TGFβRI in HSCs.	28823564	no data	
let-7e	1.53	−1.45 *	miRNA-99b/let-7e/miRNA-125a cluster is highly-expressed in hepatocarcinoma, and its expression can be regulated by ZEB1.	30840268	up (liver)	32710190
miR-125a-5p	1.51	−1.43 *	miRNA-99b/let-7e/miRNA-125a cluster is highly-expressed in hepatocarcinoma, and its expression can be regulated by ZEB1.	30840268	no data	
miR-29c	−1.75	−1.25	Anti-fibrotic miRNA which plays a crucial role in fibrotic disease. Overexpression inhibited hepatocellular damage and liver fibrosis in mice.	28130757 30917489	down (serum)	29848284
miR-30d	−1.77	−1.09	Acts as a tumor suppressor in hepatocellular carcinoma.	27571925	no data	
miR-125b-5p	−1.81	−1.33	In humans and rodents, epigenetic silencing of miR-125b-5p upregulates ITGA8 expression to activate the RhoA signaling pathway, leading to liver fibrosis in NAFLD.	31926204	no data	
miR-194	−1.89	−1.14	miR-194 plays a protective role by inhibiting the activation and proliferation of HSCs via AKT2 suppression; miR-194 agomir treatment dramatically ameliorated liver fibrosis in CCl_4_-treated mice.	31496625	no data	
miR-30a	−1.93	1.02	Anti-fibrotic miRNA which suppresses the activation of hepatic stellate cells by inhibiting EMT. Down-regulated in the liver of patients with cirrhosis and rat liver after treatment with CCl_4_.	29587268	no data	
miR-455	−2.02	−1.32 *	miR-455-3p was significantly downregulated during HSCs activation. In addition, the reduction of miR-455-3p was correlated with liver fibrosis in mice with carbon tetrachloride (CCl_4_), bile duct ligation (BDL), and high-fat diet (HFD)-induced liver fibrosis.	31150929	no data	
miR-192	−2.14	1.34 *	Down-regulation of miR-192 in HSCs was found to be an early event during fibrosis progression in mouse models of liver injury.	26096707	down (liver) up (serum)	19030170 30142428
miR-203	−4.48	1.33	Hepatic overexpression of miR-203 can facilitate the initiation of liver regeneration and enhance the potency of liver regeneration after 70% PH in cirrhotic rat.	28501131	no data	
miR-193	−7.22	1.05	Downregulated not only in experimental hepatofibrogenesis but also in human liver fibrosis; miR-193 can modify the TGF-β-dependent regulation of extracellular matrix-related genes in HSCs in the manifestation and resolution of liver fibrosis.	26120970	no data	
**Inflammation**						
miR-106b	2.66	2.06	Over-expressed in serum of patients with hepatocarcinogenesis.	29179453	up (liver)	26733244
miR-100	2.47	−1.52 *	Increased serum level in HBV-positive small HCC compared to other benign liver diseases associated with HBV.	25519019	no data	
miR-322	2.42	1.06	PPARγ-dependent miR-424(miR-322 orthologue)/503-CD40 signaling axis is critical for regulation of inflammation mediated angiogenesis.	28566713	no data	
miR-223	1.57	−1.36	miR-223 plays a key role in controlling steatosis-to-NASH progression by inhibiting hepatic Cxcl10 and Taz expression.	30964207	up (liver)	30964207
miR-99a	−1.66	−1.54 *	Decreased expression of miR-99a is correlated with tumor differentiation, liver cirrhosis and patients’ outcome in HCC.	24732044	down (serum)	25232454
miR-26b	−1.98	−1.51 *	Hepatic expression of miR-26b-5p was decreased in the fibrotic liver, with a negative correlation to PDGFR-β and fibrosis and angiogenesis markers in mice.	30901579	no data	
miR-365	−2.02	−1.21	miR-365 was downregulated in hepatocellular carcinoma and inhibited HCC cell proliferation and invasion.	30182377	no data	
miR-22	−2.04	−1.3	Immunosuppressive microenvironment promoted by HSC-derived galectin-1 in HCC can be inhibited by miR-22.	27494859	no data	
miR-378	−2.36	−1.02	MicroRNA-378 promotes hepatic inflammation and fibrosis via modulation of the NF-κB-TNFα pathway.	30218679	no data	
**Metabolism**						
miR-132	12.85	1.04	miR-132 as a key regulator of hepatic lipid homeostasis.	28381526	up (serum)	32443971 28381526
miR-152	4.13	−1.08	Anti-proliferative and pro-apoptotic roles in hepatocarcinogenesis.	30967300	no data	
miR-93	4.07	−1.05	Over-expressed in serum of patients with hepatocarcinogenesis.	29179453	no data	
miR-15b	3.42	−1.22	Incresed serum expression in patients with early hepatocellular carcinoma associated with hepatitis B virus.	26264553	up (serum)	23287814
miR-181a	1.93	−1.58 *	Profibrotic role of miR-181b in HSC activation and miR-181b activation of HSCs occurs via PTEN/Akt pathway.	25148875	up (serum)	30558790
miR-28	1.6	−1.2	MicroRNA-28-5p regulates liver cancer stem cell expansion via IGF-1 pathway.	31885628	up (serum)	20497147
miR-122	−1.5	1.37 *	Highly abundant expression in liver; involved in lipid metabolism, iron homeostasis and differentiation of hepatocytes, reduced hepatic level in NASH patients; important role in hepatocyte survival and tumor suppression.	25537773 19030170 22820290	down (liver) up (serum)	19030170 30142428
**Miscellaneous**						
miR-290	39.2	−1.25	Pro-survival function for the mir-290-295 cluster in mouse embryonic stem cells.	21573140	no data	
let-7f	−1.51	−1.58 *	Decreased expression after 50% partial hepatectomy in rats.	21183868	no data	

Values are given as mean; * = *p* < 0.05 CCl_4_ PDE5 inhibitor versus CCl_4_ vehicle.

## Data Availability

The data presented in this study are available in Supplemental Tables and the data presented in this study are available on request from the corresponding author.

## Supplementary Materials

The following are available online at https://www.mdpi.com/1422-0067/22/1/382/s1, Figure S1. Principal component analysis of miRNA expression profiling results. Displayed are the first three major components from the principal component analysis. Figure S2. Correlation of ALT level with plasma exosomal miRNA expression. Fold change of plasma exosomal miRNA expression of control, CCl_4_/Vehicle and CCl_4_/PDE5 inhibitor treated rats were correlated with ALT level. Spearman’s correlation coefficient and corresponding P values are indicated. Figure S3. Schematic overview of the experimental design. Table S1. Up-regulated hepatic mRNA expression in CCl_4_ treated rats compared to control animals. Table S2. Down-regulated hepatic mRNA expression in CCl_4_ treated rats compared to control animals.
