# Supplementary material for: Exosomal miRNAs as Potential Biomarkers to Monitor Phosphodiesterase 5 Inhibitor Induced Anti-Fibrotic Effects on CCl4 Treated Rats
Source: Int J Mol Sci. 2020 Dec 31;22(1):382. doi: 10.3390/ijms22010382 (PMC7795540; doi:10.3390/ijms22010382)
Supplement: Supplementary file 1 [file ijms-22-00382-s001.zip › ijms-1036920-supplementary/ijms-1036920-supplementary.docx]

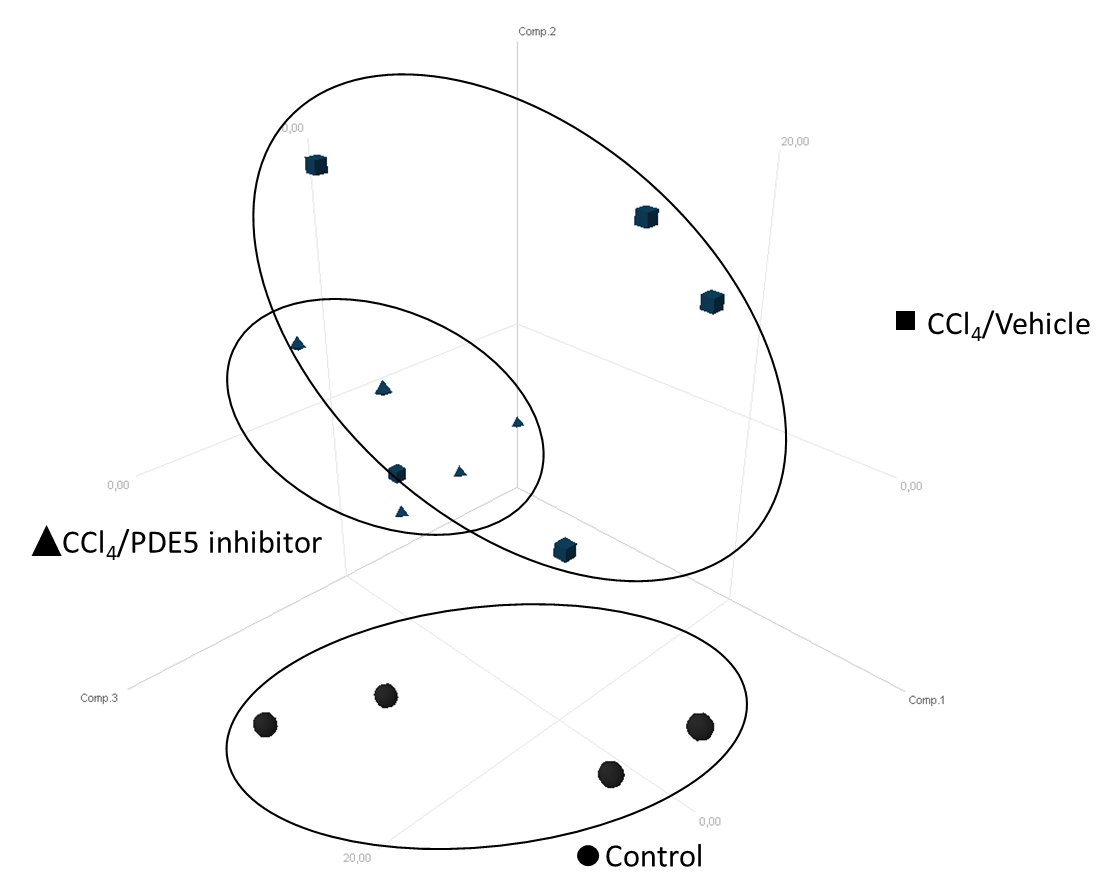


**Figure S1.** Principal component analysis of miRNA expression profiling results. Displayed are the first three major components from the principal component analysis.

Control

CCl_4_/Vehicle

CCl_4_/PDE5 inhibitor

**Figure S2.** Correlation of ALT level with plasma exosomal miRNA expression. Fold change of plasma exosomal miRNA expression of control, CCl_4_/Vehicle and CCl_4_/PDE5 inhibitor treated rats were correlated with ALT level. Spearman´s correlation coefficient and corresponding P values are indicated.

**
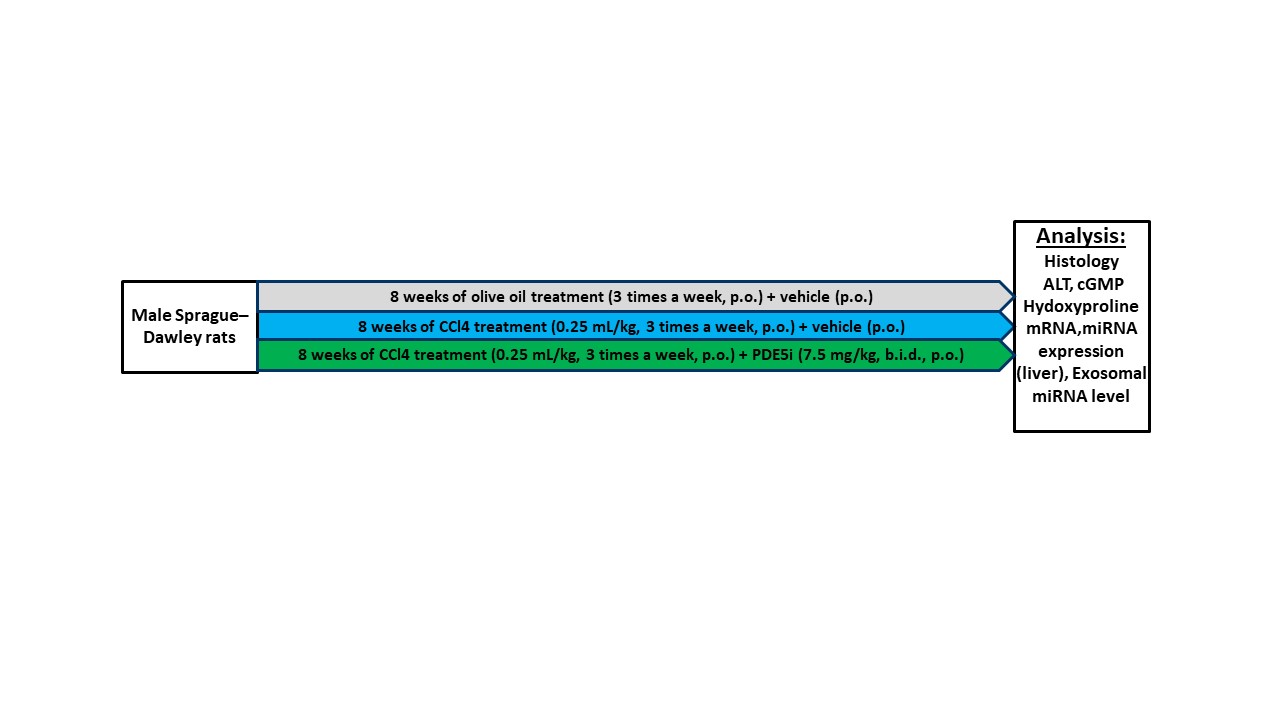
Figure 3.** Schematic overview of the experimental design.
